# Pertussis in high-risk groups: an overview of the past quarter-century

**DOI:** 10.1080/21645515.2020.1738168

**Published:** 2020-04-16

**Authors:** Victoria A Jenkins, Miloje Savic, Walid Kandeil

**Affiliations:** a Vaccines, GSK, Wavre, Belgium

**Keywords:** Pertussis, high risk, asthma, COPD, smoking, obesity

## Abstract

Infectious diseases can impact chronic medical conditions. However, it is currently not clear how pertussis correlates with preexisting or underlying disorders. We reviewed literature from the last 25 years to describe the burden and impact of pertussis infection in specific risk groups in individuals aged ≥11 years.

Our literature search returned 543 hits, of which 18 were eligible for this review. Adolescents and adults with underlying conditions, such as asthma, chronic obstructive pulmonary disease (COPD), or obesity are potentially at increased risk of pertussis infection. Immunodeficiency and smoking have also been associated with worsened pertussis symptoms and an increased pertussis-related hospitalization rate. In patients with pertussis and preexisting asthma or COPD, symptoms were worsened, and health-care costs were consequently increased.

Further efforts are needed to close the knowledge gap and to understand the burden of pertussis in at-risk adolescent and adult populations to help inform vaccination strategies and recommendations.

## Background

Chronic diseases, as well as general medical conditions, can be exacerbated by different infectious diseases.^1–5^ Since life expectancy in developed countries is rapidly increasing, predominantly due to a lower mortality rate among older individuals,^6,7^ the prevalence of age-related chronic diseases is predicted to increase, along with healthcare-associated costs.^8^ In addition, multi-morbidity is known to increase with age.^8^ For instance, a cross-sectional study assessing the prevalence of 40 morbidities in 1.7 million individuals from Scotland, UK, showed that at least two chronic medical conditions were present in 30.4% of adults aged 45–64 years, in 64.9% of those aged 65–84 years, and in >80% of >85 year-olds.^9^ Prevention of infectious diseases in all age groups, especially older adults, could, therefore, contribute to maintaining or improving quality-of-life by limiting exacerbations of underlying medical conditions.

Pertussis, commonly known as “whooping cough,” is an acute infectious and highly contagious disease that affects people of all ages, and is caused by the bacterium *Bordetella pertussis*.^10^ Since the introduction of effective vaccines in the 1940s, pertussis incidence has, for example, steadily decreased in the United States (US) by more than 80% among all age groups.^11^ Neither natural immunity due to previous infection nor vaccination provides life-long protection; thus, despite the high infant pertussis vaccination coverage rates, there has been a global resurgence of the disease in developed regions in adolescents and adults.^12–14^ Besides waning of immunity and other previously published contributing factors,^13–17^ absent or inconsistent recommendations for booster vaccinations in adults, and thus low vaccine coverage in specific populations or regions, may also significantly add to the recent resurgence. Consequently, pertussis booster vaccination throughout life is important to help prevent pertussis infection and its transmission in those at risk of infection.

With respect to pertussis vaccination, in the US it is recommended that children should receive a diphtheria-tetanus-acellular pertussis (DTaP) containing vaccine at 2, 4, 6 months, between 15 and 18 months, and at 4–6 years. A booster dose of a reduced antigen content tetanus-diphtheria-pertussis (Tdap) vaccine is recommended for those 11 or 12 years of age and for any adult 19 years of age or older who has never received a dose of Tdap.^18^ The Advisory Committee on Immunization Practices recently updated their recommendation for adults to receive one dose of either a Td or Tdap vaccine every 10 years. In addition, pregnant women should receive one dose of Tdap during each pregnancy. Despite these recommendations in the US, uptake of pertussis-containing vaccines in adults is still well below target.^19^ In addition, adult recommendations vary globally, for example, the UK does not have an adult pertussis vaccination recommendation except for vaccination during pregnancy.^20^

*B. pertussis* infection has a clinical presentation ranging from a relatively mild cough illness to a severe and potentially fatal illness with pneumonia, seizures, encephalopathy, and respiratory failure.^12^ While most severe and fatal pertussis cases occur in infants, pertussis infection can also have serious manifestations in older children and adults.^21,22^ Similarly, most pertussis-related hospitalizations occur in infants,^23,24^ although up to 12% of adults with pertussis may also require hospitalization.^25^ In fact, the true burden of pertussis disease in adults is unknown, with cases in adults being frequently missed or misdiagnosed.^13,26,27^ A high proportion of adults hospitalized for pertussis have underlying medical conditions,^24,28^ suggesting that these may be a contributing factor to pertussis disease severity. Between 1990 and 2004, the US. Centers for Disease Control and Prevention received 5 reports of pertussis-associated deaths in adults (aged 49–82 years), all of whom suffered from comorbid conditions, such as diabetes, multiple sclerosis with asthma, multiple myeloma (requiring immunosuppressive therapy), myelofibrosis, and chronic obstructive pulmonary disease (COPD).^12^ Thus, it is important to ascertain which patients are at increased risk of pertussis disease (and related complications) and whether they could benefit from targeted prevention strategies.

Few data exist on the current burden and characteristics of severe pertussis disease in adolescents and adults.^24^ A recent review paper provided a detailed overview of the disease burden and the role of vaccination in older adults.^26^ Here we have reviewed the literature from the last 25 years to describe the current knowledge on the burden and impact of pertussis infection in specific risk groups in individuals aged ≥11 years, an age beyond which pertussis vaccination recommendations are variable and uptake is below the rates seen for pediatric pertussis-containing vaccines.

## Methods

### Search strategy

A search was performed in PubMed on July 17, 2019 to identify papers related to pertussis infections in different risk groups as well as the burden of disease associated with underlying conditions in these groups. The string used in our search is provided in the supplementary material. The studies related to children <11 years, diagnostic, molecular and immunology, outcome of infection, mechanisms of infection, or reviews were excluded.

## Results

### Study selection

**Figure 1.**
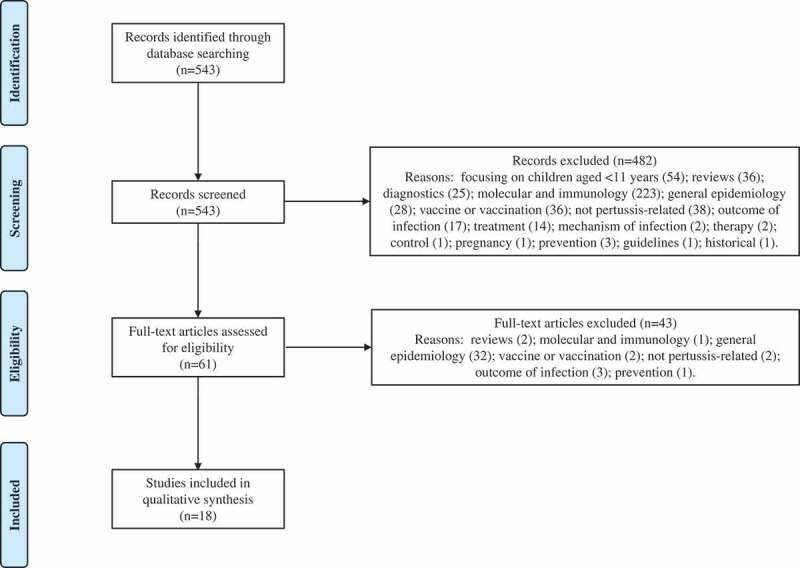
Flow diagram of studies included in the review n, number of records included in each step of review

Our search returned 543 results. Based on the screening of titles, abstracts, or main texts, 482 were excluded. Sixty-one full-text articles were further screened, of which 18 were included in this review. Articles were excluded for the following reasons: focusing on children aged <11 years (54), reviews (38), diagnostics (25), molecular and immunology (224), general epidemiology (60), vaccine or vaccination (38), not pertussis-related (40), outcome of infection (20), treatment (14), or based on other reasons (12). A flow diagram showing the inclusion/exclusion of studies is presented in Figure 1. Eight case studies appeared in our search, and whilst they are not representative of the general population, they do provide further support of severe or atypical pertussis diagnosis in patients with preexisting or underlying conditions.

### Underlying conditions associated with an increased risk of severe pertussis in adults

**Figure 2.**
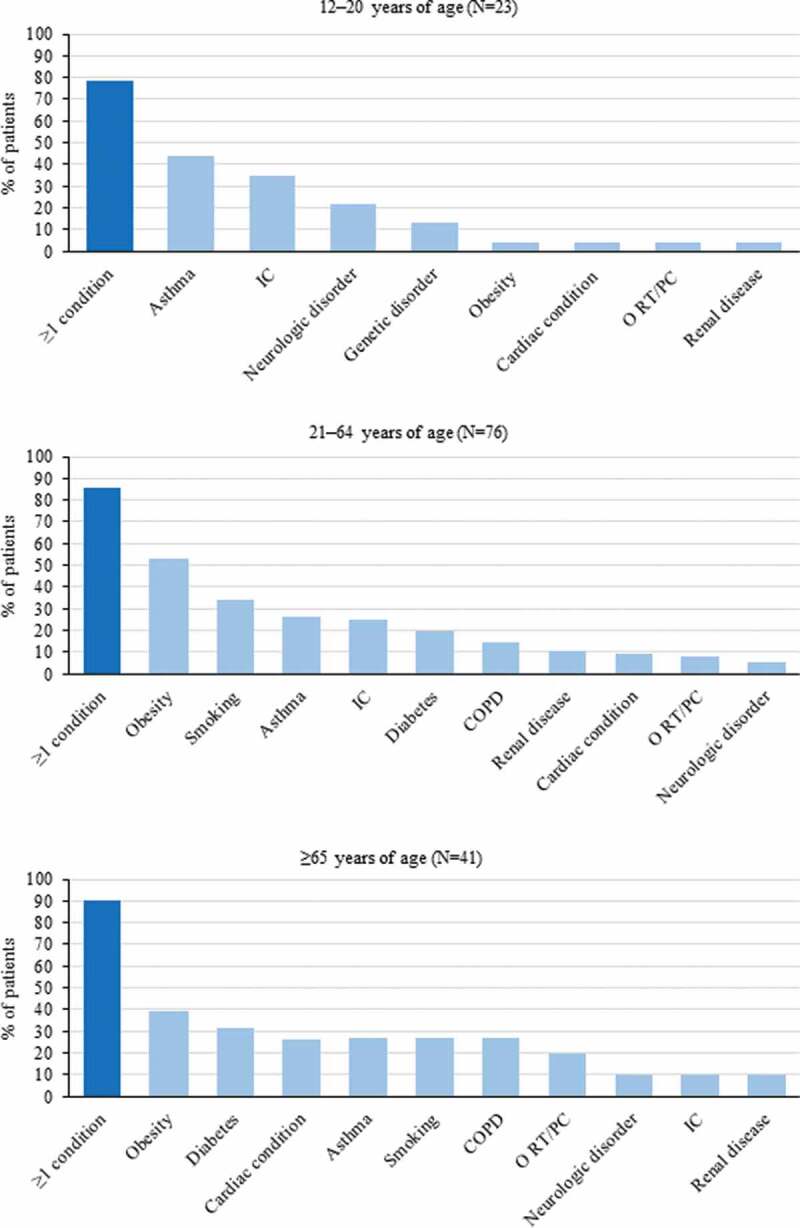
The most frequent underlying medical conditions in hospitalized pertussis patients by different age groups (Mbayei, Clin Infect Dis 2018)^24^ IC, immunocompromising condition; N, number of hospitalized pertussis patients; O RT/PC, other respiratory tract/pulmonary condition; COPD, chronic obstructive pulmonary disease

Enhanced Pertussis Surveillance and inpatient medical record data collected via the US. Emerging Infections Program Network between 2011 and 2015 showed that among 15,942 reported pertussis cases (mean annual incidence: 20.3 per 100,000 persons), 3.2% required hospitalization. Of these, 29.6% of cases and 4.5% of hospitalizations occurred in 12–20-year-olds, 18% of cases and 14.8% of hospitalizations in 21–64-year-olds, and 2.1% of cases and 8.3% of hospitalizations in >65-year-olds. Of the hospitalized patients, 32.6% had underlying conditions, with the highest proportion (87.2%) observed among adults aged ≥21 years. The most frequent conditions in 12–20–year-olds were asthma, immunocompromising conditions, and the use of immunocompromising medication. Obesity and smoking (current or former) were most frequent in 21–64-year-olds, and asthma and cardiac conditions in ≥65-year-olds (Figure 2). Of the three hospitalized patients who died, one was an infant aged 42 days and two were adults with underlying medical conditions: one 48-year-old adult who had a history of human immunodeficiency virus and one 76-year-old adult who had a previous history of asthma and COPD.^24^

#### Asthma

**Figure 3.**
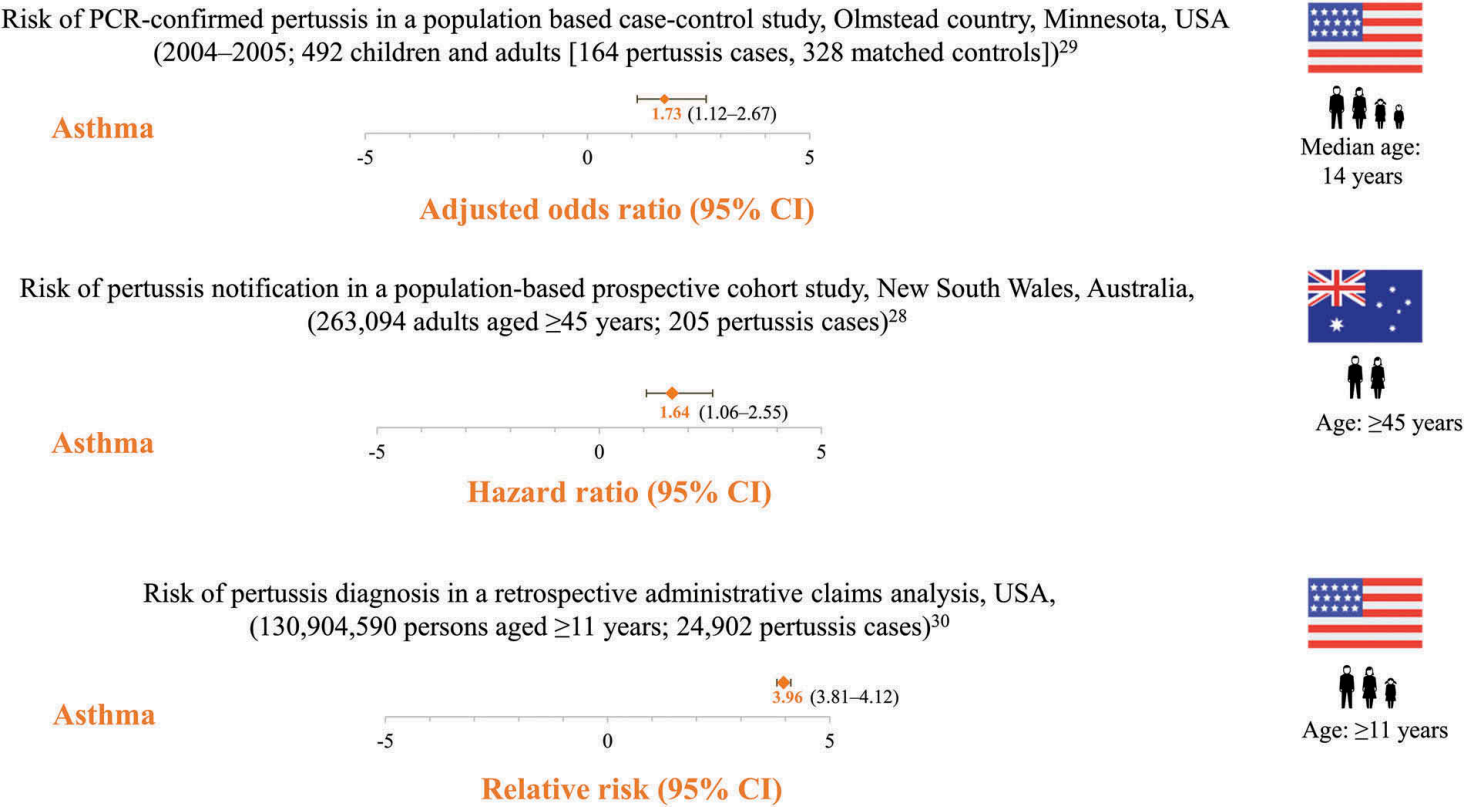
Increased risk of pertussis in patients with asthma PCR, polymerase chain reaction; CI, confidence interval

Three studies have shown that individuals with underlying asthma are at increased risk of pertussis diagnosis compared to individuals without, as further illustrated by two case studies (Figure 3).^28–32^ A retrospective, population-based case–control study compared the history of asthma in children and adults who had pertussis (confirmed by polymerase chain reaction, PCR) with age- and sex-matched controls during a pertussis outbreak between 2004 and 2005 in California, USA. Of the 164 cases, 38% had asthma before the pertussis diagnosis compared to 26% of the 328 controls, resulting in an adjusted odds ratio of 1.73 (95% confidence interval [CI]: 1.12–2.67, *P* = .013). The population-attributable risk of pertussis in asthma patients was 17%.^29^ In a study between 2006 and 2008, 263,094 adults aged ≥45 years (mean 62.8 years) were recruited in New South Wales, Australia, in a prospective, population-based cohort study and followed-up for laboratory-confirmed pertussis (PCR or culture) occurrence. Among the 205 cases, a history of preexisting asthma was a significant predictor of pertussis incidence, with a hazard ratio of 1.64 (95%CI: 1.06–2.55) for pertussis in individuals with asthma compared to those without.^28^ A retrospective analysis using administrative claims data in the United States evaluated the incidence of pertussis among 1,041 adolescents and adults aged >11 years with preexisting asthma and a matched cohort consisting of 1,041 pertussis patients without preexisting asthma. The incidence of pertussis among patients with preexisting asthma was 0.27 per 1000 person-years (95%CI: 0.27–0.29), resulting in a relative risk of pertussis of 3.96 (95%CI: 3.81–4.12) when compared to controls. The risk was highest among 19–64-year-olds with preexisting asthma (relative risk 4.06 [95%CI: 3.86–4.27]).^30^

During the 1998 pertussis outbreak in Quebec, Canada, positive cases (either culture-positive or meeting the Canadian surveillance case definition for pertussis and having no other apparent cause) were evaluated in 280 patients aged 12–17 years and in 384 patients aged ≥18 years. Respectively, 24% and 13% had asthma. The authors observed higher asthma prevalence in pertussis patients compared to the general adolescent population of Canada, which was estimated to be ~12%. In pertussis patients with asthma, the mean duration of paroxysmal cough (5 vs. 4 weeks, *P* = .004), risk of sinusitis (16% vs. 12%, *P* = .4) and mean number of nights disturbed by pertussis (26 vs. 20 nights, *P* = .03) were also increased compared to pertussis patients who did not have asthma.^33^

Among individuals hospitalized for pertussis, approximately 44% of 12–20-year-olds and approximately 26% of ≥21-year-olds had asthma, proportions that are considerably higher than the prevalence of asthma in the general US. population, estimated at 10% of the adolescent and 8% of the adult population.^24^ Asthma (and/or COPD) exacerbations were among the most frequent diagnoses leading to pertussis-related hospitalizations in both adolescents and adults.^24^ An increased all-cause and pertussis-related hospital admission rate was also observed for pertussis patients with asthma compared to controls at 45 days, 3 months, or 6-months post-pertussis diagnosis.^30^

In a case–control study in Finland in 1999, involving 103 adults with stable asthma (53 mild, 50 moderate) and 30 healthy controls, *B. pertussis* was detected by PCR in the sputum of 28.3% of mild asthmatics, in 20.0% of moderate asthmatics, and in 16.7% of controls. Asthma symptom scores were above the median in 66% of *B. pertussis*-positive asthmatics compared to 47% in *B. pertussis-*negative asthmatics. In addition, as shown by the forced expiration volume in 1 s/forced vital capacity, lung function was lower in asthmatics with *B. pertussis* compared to those without (77.1% vs. 80.7%).^34^

In pertussis patients with or without asthma, health-care costs adjusted to 2014 US. dollars using the medical care component of the US. Consumer Price Index were also assessed.^35^ Within 45 days of pertussis diagnosis, pertussis patients with asthma accrued 2,007 USD in all-cause adjusted health-care costs (medical and pharmacy), whereas matched pertussis patients without asthma accrued 814 USD resulting in 1,193 USD more in all-cause adjusted costs. This difference increased to 1,301 USD in the 3 month post-pertussis diagnosis and to 1,639 USD in the 6 month post-diagnosis. Within 45 days of pertussis diagnosis, the greatest difference ((2,510 USD)) was seen in ≥65-year-olds while the smallest (746 USD) in 11–18-year-olds.^30^ The difference in pertussis-related adjusted costs between pertussis patients with or without asthma was 230 USD within 45 days of pertussis diagnosis, 241 USD within 3 months, and 251 USD within 6 months, and was similar between all age groups.^30^

#### COPD

Two studies have shown that individuals with COPD are potentially at increased risk of pertussis diagnosis or pertussis-related hospitalizations compared to adults without COPD.^24,30^ Emerging Infections Program Network data collected between 2011 and 2015 showed that COPD was more frequent among hospitalized adult pertussis patients (19%) compared to the proportion of adults in the United States with a history of COPD (6.4%).^24^ COPD exacerbations were among the most frequent diagnoses leading to pertussis-related hospitalizations in adults.^24^ In a retrospective administrative claims analysis, the relative risk of pertussis in individuals aged ≥11 years with preexisting COPD was over two times higher than in matched controls (relative risk 2.53 [95%CI: 2.40–2.68]), with the highest relative risk (3.59 [95%CI: 3.35–3.84]) observed among patients aged 19–64 years.^30^ Within 45 days, 3 months, and 6 month post-pertussis diagnosis, all-cause hospitalization rates were higher in pertussis patients with COPD than in those without compared to the pre-diagnosis date. COPD patients were almost two times more likely to be hospitalized due to pertussis across all three periods evaluated.^30^ Health-care costs were also assessed in pertussis patients with or without COPD. The accrued adjusted health-care cost (medical and pharmacy) per pertussis patient with COPD was 4,751 USD and 1,057 USD per matched pertussis patient without COPD, resulting in 3,694 USD difference in all-cause adjusted costs within 45 days of pertussis diagnosis. This difference increased to 6,154 USD in the 6 month post-pertussis diagnosis for the patients with pertussis and COPD.^30^

In a case–control study comparing seroprevalence of *B. pertussis* in 90 patients with COPD and 90 age- and sex-matched controls with other lung diseases who attended a tertiary care hospital in Hamedan, Iran, a statistically significant association was found between COPD and anti-pertussis toxin (PT) immunoglobulin (Ig) G seropositivity, noting that the presence of IgG antibodies in study participants is indicative of past infection(s). However, an association between *B. pertussis* seroprevalence and severity of COPD was not shown.^36^ These results should be interpreted taking into account that the threshold for seropositivity was selected arbitrarily and that there is no international threshold indicative for previous pertussis infection. Some inconsistencies can also be noted in the assessments made by COPD severity. Overall, the authors hypothesized that the colonization by *B. pertussis* could be a potential risk factor for the development of COPD.^36^

#### Chronic bronchitis

In an observational study between 2000 and 2002 in Basel, Switzerland, the occurrence of *B. pertussis* and *B. parapertussis* infection was evaluated in 26 patients (age: median 71, range 34–86) diagnosed with acute exacerbations of chronic bronchitis. Nasopharyngeal swabs from all 26 participants were negative by both culture and PCR. However, serology, using an arbitrary seropositivity threshold, indicated *B. pertussis* infection in 19%, and dubious *B. parapertussis* or *pertussis* infection in 12% of cases.^37^

#### Smoking

Enhanced Pertussis Surveillance and inpatient medical record data collected between 2011 and 2015 showed that among ≥21-year-olds hospitalized with pertussis, 31.6% were current/former smokers.^24^ (In perspective, 15.7% of the US. adults ≥18 years of age were current cigarette smokers in 2016^38^).

Two other studies also support the hypothesis that smoking could contribute to the worsening of pertussis symptoms.^33,39^ During the 1998 pertussis outbreak in Quebec, Canada, the mean duration of paroxysmal cough (5 vs. 4 weeks, *P* = .004), risk of sinusitis (19% vs. 11%, *P* = .008), and mean number of nights disturbed by pertussis (25 vs. 20 nights, *P* = .4) were increased in smokers compared to nonsmokers aged ≥12 years.^33^ A population-based prospective cohort study in Australian adults aged ≥45 years found no association between smoking and the occurrence of pertussis.^28^ Nevertheless, a nested case–control study suggests that smoking increases the risk for severe pertussis disease, as shown by the increased rate of pertussis-related hospitalizations in smokers compared to nonsmokers aged ≥45 years (odds ratio 2.37 [95%CI: 1.11–5.06]).^39^

#### Obesity

Data from the US. Emerging Infections Program Network collected between 2011 and 2015 show that 47.9% of ≥21-year-old pertussis patients were obese.^24^ (In perspective, 38% of the general US. population are obese^38^). In a population-based prospective cohort study in Australia, a high body mass index (BMI) was also associated with an increased pertussis risk in ≥45-year-olds (relative risk 1.52 [95%CI: 1.06–2.19] for a BMI of 30+ kg/m^2^ vs a BMI of <25 kg/m^2^).^28^ However, in the population-based nested case–control study evaluating hospitalized pertussis cases, the association between obesity and pertussis-related hospitalization was not statistically significant, although the point estimate for the adjusted odds ratio (2.26 [95%CI: 0.89–5.74]) suggests that individuals with a BMI ≥30 may be at increased risk of being hospitalized due to pertussis infection.^39^

#### Immunocompromising conditions

Among adult pertussis patients aged ≥21 years included in the US. Emerging Infections Program Network, a higher prevalence of immunocompromising conditions was observed compared to the general adult population in the United States: 19.7% vs. approximately 3%.^24,40^ Even though the prevalence of immunocompromising conditions was relatively low (6.8%) when considering all ages (<2 months to ≥65 years), the prevalence was 34.8% in 12–20-year-olds. Taken together with the frequent underlying neurologic or genetic disorders in 12–20-year-olds participating in the study, these data suggest that many of the few adolescents hospitalized for pertussis have complex medical histories, which might explain the higher rate of pertussis-related complications in this age group, such as new-onset seizures and encephalopathy.^24^

Six case studies reported mild to severe pertussis disease in adults aged 31–82 years with underlying conditions as Diabetes Mellitus Type II, Wegener’s granulomatosis, multiple myeloma, renal transplantation, and acquired immune deficiency syndrome.^41–46^

#### Mannose-binding lectin deficiency (MBL)

One study evaluated the risk of pertussis in patients with MBL. Among study participants of all ages (median age of 15 years, age range: 1–71), severe MBL deficiency was significantly more frequent among the 125 pertussis patients than among the 430 controls (11.2% vs. 5.8%, odds ratio 2.0 [95%CI: 1.0–4.1]). Severe MBL deficiency was detected in 20.4% and 8.6% of adults (>18 years of age) with and without pertussis, respectively (odds ratio 2.7 [95%CI: 1.1–6.5]).^47^

## Discussion

Several studies have shown that underlying diseases may increase the risk of pertussis diagnosis. In particular, our literature search identified studies focusing on asthma, COPD, and obesity. Smoking is a risk factor for illnesses like COPD or lung cancer and it also appears to increase the risk of pertussis diagnosis.

Asthma is the most frequent chronic medical condition during childhood and is also a major cause of morbidity in adults. Its prevalence increased in the United States between 2001 and 2010 and appeared to start plateauing in the mid-2010s.^48,49^ In 2017, according to the World Health Organization, 235 million people were suffering from asthma worldwide.^50^ Bacterial organisms, such as *Streptococcus pneumoniae, Haemophilus influenzae*, and *Moraxella catarrhalis*, have been demonstrated to be clinically relevant contributors to asthma exacerbations.^51^ In addition, studies have demonstrated an increased risk of invasive or serious pneumococcal diseases among adults with asthma and subsequently updated recommendations by the Advisory Committee on Immunization Practices indicate that adults with asthma should receive a single dose of 23-valent pneumococcal polysaccharide vaccine to prevent serious pneumococcal infections.^52^ Thus, bacterial organisms are an important consideration in the clinical management of patients with asthma. However, to-date, there is no consensus as to whether individuals with preexisting asthma have an increased risk of pertussis infection.

Studies included in this review, covering >130 million individuals aged ≥11 years and >25,000 pertussis cases indicate that adolescents and adults with asthma are at an increased risk of pertussis and pertussis-related hospitalizations compared to individuals without asthma.^28–30^ Asthma may also be worsened in individuals with pertussis, as reflected by a reduction in lung function,^34^ worsening of symptoms,^33^ and an increase in pertussis-related hospitalizations.^24,30^ In addition, one study demonstrated that all-cause and even pertussis-related health-care expenses are higher in pertussis patients with asthma than in those without.^30^

COPD is a chronic inflammatory disease and usually progressive conditions are associated with an irreversible decline of pulmonary function, a substantial deterioration of quality-of-life, and an increase in the use of health-care resources. It is also anticipated that COPD will become the third leading cause of deaths worldwide by 2030.^53^ The complexity of COPD is reflected by the transient and apparently stochastic nature of acute exacerbations, when additional medical treatment and even hospitalization are usually required.^54^ However, the most significant issue in patients with COPD is respiratory system dysbiosis which increases susceptibility to bacterial and viral infections that could trigger acute COPD exacerbations.^55,56^

The association between COPD and pertussis in clinical settings has not been investigated extensively, and as such the overall burden of pertussis in COPD patients is unclear. Since we conducted our search in Pubmed, an additional study was published describing the occurrence of respiratory pathogens in nasopharyngeal aspirate samples from adults admitted with acute exacerbation of COPD (AECOPD) or asthma exacerbation. Interestingly, *B. pertussis* was detected in patients with AECOPD, although the sample size was limited and did not allow for a comparison.^57^ Two studies included in this review have also shown, albeit in a non-definitive manner, an increase in the risk of pertussis diagnosis and pertussis-related hospitalizations in patients with COPD, as compared to patients without COPD.^24,30^ In addition, another study found a correlation between IgG antibodies against *B. pertussis* and COPD, which could indicate a persistent subclinical infection.^36^ All-cause costs and pertussis-related health-care expenses were also higher in pertussis patients with COPD than in those without,^30^ suggesting an additional cost to the healthcare system of managing a patient with both COPD and pertussis. In contrast, non-typeable *Haemophilus influenzae* (NTHi) and *Moraxella catarrhalis* were the most frequently identified bacteria in sputum collected from 127 COPD patients aged 40–85 years collected at acute exacerbations.^58^ Among the subset of patients with bronchiectasis, *Haemophilus* was the dominant genus observed both in stable state and exacerbation events. The same study also failed to show an association between COPD and *B. pertussis* infection.^58^ We note however several limitations that may have potentially impacted the detection of *B. pertussis* in COPD patients in this study. Most importantly, assessments were only performed on sputum samples, which might not be optimal for the detection of *B. pertussis* infection, especially if collected after initiation of antibiotic or oral corticosteroid treatment for an exacerbation.^58,59^ As such, further investigation is required to understand the possible impact of pertussis infection in patients with COPD.

Smoking is accepted to be the highest risk factor for the development of COPD, while chronic bronchitis can affect between 14% and 74% of all patients with COPD.^60^ Targeted and early disease prevention strategies may, therefore, benefit patients with COPD. Studies identified by our literature search show that a relatively high proportion of individuals hospitalized with pertussis were smokers^24,39^ and that smoking was associated with the worsening of pertussis symptoms.^33^ However, no association between smoking and occurrence of pertussis was found in the study by Liu et al.^28^ A high *B pertussis* seroprevalence was demonstrated among 26 chronic bronchitis patients,^37^ although the small sample size and locational homogeneity of study participants, arbitrary selection of seropositivity thresholds and classification of cases, and lack of PCR or culture confirmation of any seropositive cases may hamper the generalizability of these findings.

Pertussis-related fatalities were reported for adults with a history of immunodeficiency, asthma, and COPD, and in other immunocompromised patients.^24,41,44^ This is consistent with reports received by the US. Centers for Disease Control and Prevention between 1990 and 2004, when five adults aged 49–82 years with serious underlying medical conditions (e.g. severe diabetes, severe multiple sclerosis with asthma, multiple myeloma on immunosuppressive therapy, myelofibrosis, and COPD) died due to pertussis.^41^

Obesity was also identified as a potential risk factor for severe pertussis disease.^24,61^ Although the association between obesity and pertussis-related hospitalization was found not to be significant, a point estimate for the odds ratio suggests that hospitalization risk may be increased.^39^

Our search also identified one article showing a relation between pertussis and MBL deficiency,^47^ which is a relatively common condition and which affects the immune system in 5–30% of people. This condition is characterized by low levels of MBL in the blood, which increases the risk of recurrent infections.^62^

Many of the articles reviewed herein face similar limitations. Whether the observed associations between underlying conditions and *B. pertussis* infection are a result of increased awareness and a higher likelihood of these individuals to seek medical attention or a predisposition for a more severe disease is not clear. In addition, the increased pertussis-related hospitalization rates in individuals with underlying medical conditions might be confounded by the perceived risk of disease, which is higher in such populations. Although outside the direct scope of this review, it is also important to note that the exact mechanism by which underlying diseases influence the systemic response to an infection is not fully understood.^63^ It is assumed that when underlying conditions are present, the organism reacts with a weak local and systemic inflammatory response to pathogens,^64^ which might explain the increased risk of infection and exacerbation of underlying diseases.^55,56^ Future observational studies are therefore needed to unravel the mechanism by which underlying disorders interfere with infections. However, trials involving underlying conditions are difficult to set up since the heterogeneity of acute and/or chronic conditions affect the reproducibility of results.

## Conclusion

Adolescents and adults with underlying conditions, such as asthma, COPD, or obesity are potentially at increased risk of pertussis infection or exacerbation. In addition to these, immunodeficiency and smoking have also been associated with worsened pertussis symptoms and an increased pertussis-related hospitalization rate. In patients with pertussis who had preexisting asthma or COPD, symptoms were worsened and health-care costs were consequently increased. An improved understanding of pertussis infections and clinical pathway for those at risk of complications from pertussis will help guide prevention, control, and treatment strategies. Thus, further studies are needed to establish the true burden of pertussis in adolescent and adult populations and to formulate a conclusive vaccination strategy on an international level to help protect those at risk.

**Figure 4.**
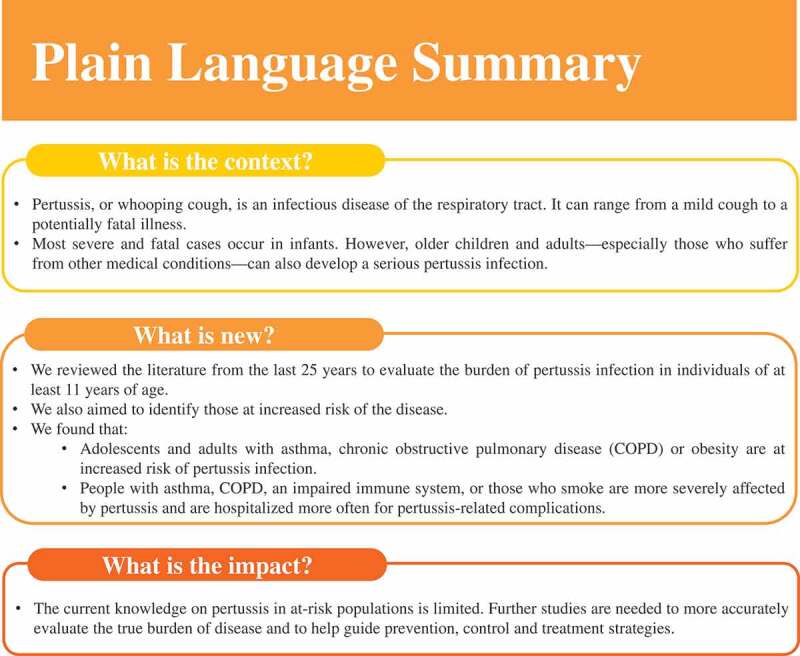
Plain language summary

A plain language summary contextualizing the results and potential clinical research relevance and impact is presented in Figure 4.

## Supplementary Material

Supplemental Material
