# Supplementary material for: Pertussis in high-risk groups: an overview of the past quarter-century
Source: Hum Vaccin Immunother. 2020 Apr 16;16(11):2609–17. doi: 10.1080/21645515.2020.1738168 (PMC7746252; doi:10.1080/21645515.2020.1738168)
Supplement: Supplemental Material [file KHVI_A_1738168_SM4781.docx]

**Supplemental online material**

Search string:

((((((((((((pertussis[Title/Abstract] OR "whooping cough"[Title/Abstract])) AND (high-risk[Title/Abstract] OR "high risk"[Title/Abstract] OR severe[Title/Abstract] OR immunocompromised[Title/Abstract] OR immunocompromised[Title/Abstract] OR copd[Title/Abstract] OR asthma[Title/Abstract] OR hospitalised[Title/Abstract] OR hospitalized[Title/Abstract] OR "cystic fibrosis"[Title/Abstract] OR cardiac[Title/Abstract] OR myeloma[Title/Abstract] OR "Chronic bronchitis"[Title/Abstract] OR granulomatosis[Title/Abstract))) NOT (Animal[Title/Abstract] OR pig[Title/Abstract] OR Drosophila[Title/Abstract] OR mouse[Title/Abstract] OR rat[Title/Abstract] OR rats[Title/Abstract] OR mice[Title/Abstract] OR mouse[Title/Abstract])) NOT (maternal[Title] OR antenatal[Title] OR neonate[Title] OR neonatal[Title] OR new-born[Title] OR newborn[Title] OR infant[Title] OR infants[Title] OR pregnant[Title] OR pregnancy[Title] OR children[Title] OR childhood[Title] OR pediatric[Title] OR vaccine[Title] OR vaccination[Title] OR paediatric[Title] OR immunisation[Title] OR immunization[Title]))) AND English[Language]))))) AND ("1994"[Date - Publication] : "2019"[Date - Publication])
